# Patient-specific management in high-risk revision total hip arthroplasty: mid-term outcomes of a next-generation constrained acetabular liner

**DOI:** 10.1007/s00402-026-06527-6

**Published:** 2026-10-20

**Authors:** Farouk Khury, Blaine Luebbering, Garrett Ruff, Laith Bahlouli, Anzar Sarfraz, Ran Schwarzkopf, Vinay K. Aggarwal

**Affiliations:** 1 The Ruth and Bruce Rappaport Faculty of Medicine, Technion – Israel Institute of Technology, Haifa, Israel; 2 Department of Orthopedic Surgery, New York University Langone Medical Center, New York, USA; 3 Division of Orthopedic Surgery, Rambam Health Care Campus, Haifa, Israel

**Keywords:** Constrained acetabular liner, Constrained liner, Hip instability, THA dislocation, Implant dislocation, Prosthetic dislocation

## Abstract

**Background:**

Managing recurrent instability in revision total hip arthroplasty (rTHA) requires a personalized approach, as traditional constrained liners often yield suboptimal results due to locking-mechanism failure and limited impingement-free range of motion. This study aimed to evaluate the mid-term clinical outcomes of a novel constrained acetabular liner (CAL) when utilized as a patient-specific solution for a high-risk cohort undergoing complex rTHA. By tailoring the constraint to individual biomechanical deficits, this next-generation device seeks to mitigate historic failure modes.

**Methods:**

We retrospectively reviewed 32 patients who underwent 37 rTHA procedures with a next-generation CAL at a single urban academic center between March 2017 and October 2023. Average follow-up was 4.7 years (range, 0.3 to 9.7 years). The most common indication for rTHA was instability (70.3%), followed by infection (16.2%), aseptic loosening (10.8%), and polyethylene wear (2.7%). Just over one third of patients (37.5%) had undergone a prior revision. Preoperative constructs included fixed-bearing (37.8%), dual-mobility (32.4%), CAL (24.3%), static spacer (2.7%), and hemiarthroplasty (2.7%).

**Results:**

In most procedures (70.3%), the CAL was implanted into a pre-existing acetabular cup, whereas 29.7% involved both cup and liner revision. Following rTHA, no cases of implant wear, breakage, acetabular cup aseptic loosening, or periprosthetic fracture were observed. Six patients (18.8%) underwent a total of sixteen subsequent reoperations after CAL implantation—10 for instability and 6 for infection. Among instability-related reoperations, 4 involved revision to a new next-generation CAL. Survivorship free from all-cause reoperation was 83.8% at one year and 72.6% at latest follow-up.

**Conclusions:**

This study demonstrates favorable mid-term clinical outcomes and reoperation-free survivorship following complex revision THA using a novel constrained acetabular liner system. For patient cohorts suffering from severe recurrent dislocation, this next-generation CAL provides an effective, tailored tool in the surgical armamentarium by adapting to unique patient anatomy and soft-tissue compromises. Further research with larger sample sizes and extended follow-up is needed to assess long-term durability.

## Introduction

Despite the high success rate of primary total hip arthroplasty (THA), its growing popularity has inevitably led to an increasing number of postoperative complications requiring revision surgery [1–5]. Among these, prosthesis dislocation is one of the most common and challenging complications, occurring in 0.2 to 0.7% of primary THAs in some registries though other large series report rates between 1.9% and 10% [6] and in up to 9.0% of revision total hip arthroplasties (rTHA) [6–8]. In addition to compromising the functional outcomes, dislocation imposes a considerable burden on both the patient and the healthcare system [9–11].

Historically, the management of these complex cases has relied on generalized approaches. Various strategies have been developed to mitigate the risk of dislocation, including the use of dual-mobility implants and larger-diameter femoral heads [12]. Although effective in many cases, these options may be insufficient for patients with severe instability or compromised soft-tissue support. In the era of complex revisions, recognizing individual patient phenotypes—such as specific spinopelvic biomechanics and precise abductor muscle deficiencies—is paramount. For such high-risk patients, constrained acetabular liners (CALs) are often employed as a salvage solution tailored to the patient’s unique instability profile [13]. CALs work by capturing the femoral head within a polyethylene liner that extends beyond the head’s equator, thereby reducing the primary arc of motion and decreasing the likelihood of dislocation. However, first-generation constrained designs introduce their own challenges, including increased stresses that can contribute to aseptic loosening and liner failure [14, 15].

A next-generation CAL design aims to reduce the risk of dislocation and liner failure by improving stability while providing a greater range of motion before impingement [16]. This system features a highly cross-linked polyethylene liner and a modular 32-mm or 36-mm cobalt-chromium femoral head with a flat equatorial section angled at 15 degrees to the vertical axis. This unique design increases the force required to produce a femoral head dislocation—an attribute that is particularly valuable in cases of patient-specific abductor deficiency or recurrent dislocation [17–19].

Despite the promising short-term results reported for this next-generation CAL, few studies have evaluated its mid-term performance [17, 20–23]. This study aims to assess the mid-term clinical outcomes of a next-generation constrained acetabular liner in a cohort of complex revision total hip arthroplasties.

## Methods

### Study design

This was a retrospective review of 48 patients who underwent rTHA with implantation of a next-generation constrained acetabular liner (G7^®^ Freedom Constrained Liner [Zimmer-Biomet, Warsaw, IN, USA]) between March 2017 and October 2023 at a single, urban, high-volume, academic hospital. Patients were eligible for inclusion if they were ≥ 18 years of age. Out of the 48 patients identified, 16 were excluded because of missing follow-up data, specifically because they failed to return for postoperative clinical visits or lacked sufficient medical documentation after discharge. Institutional review board (IRB) approval was obtained before initiating this study: i23-00714. The decision to utilize this specific CAL was individualized based on preoperative radiographic assessment, intraoperative soft-tissue tensioning, and the patient’s unique history of instability. Although we lack a standard surgical protocol, the presence of complete abductor dysfunction or insufficiency was a primary reason to utilize a constrained liner.

### Data collection and analyses

Patients’ baseline characteristics, including age, sex, body mass index (BMI), race, smoking status, and American Society of Anesthesiologists (ASA) score, were collected from our institution’s electronic medical record (Epic^®^ Caboodle, Version 15 [Epic Systems Corporation, Verona, WI, USA]). Past surgical history, perioperative characteristics including surgical indication and implant details, and all reoperations after next-generation CAL implantation were also recorded. Because several surgeons performed the CAL implantations, specific perioperative variables such as the surgical approach, postoperative rehabilitation protocol, and thromboprophylaxis regimen varied between each surgeon and were individualized for each patient.

Categorical and ordinal variables are presented as counts with percentages, and continuous variables are represented as means ± standard deviations. Procedures were stratified into two groups, aseptic versus septic indications, and Kaplan-Meier analysis, treating each procedure as an independent observation, was used to estimate survivorship free from reoperation after CAL implantation. Survivorship was compared between groups using the log-rank test. Statistical analyses were performed using SPSS^®^ Statistics (Version 28 [IBM Corporation, Armonk, NY, USA]).

### Baseline characteristics

A total of 32 patients undergoing 37 rTHA procedures with CAL implantation were included. The mean age at surgery was 71.8 years, and the mean BMI was 27.2 kg/m^2^. The majority of patients were female (*n* = 23, 71.9%) and White (*n* = 26, 81.3%). Just over half of the 37 procedures were performed in patients with an ASA score of II (*n* = 19, 51.4%), followed by III (*n* = 14, 37.8%), and IV (*n* = 4, 10.8%). The mean follow-up duration was 4.7 years (range, 0.3 to 9.7 years) (Table 1).

**Table 1:** Baseline characteristics

Parameter	*N*= 37 procedures in 32 patients
Age at surgery (years)^a^, mean ± SD	71.8 ± 11.9
Sex, n (%)	
Male	9 (28.1)
Female	23 (71.9)
BMI (kg/m^2^)^a^, mean ± SD	27.2 ± 5.0
Race, n (%)	
White	26 (81.3)
Black or African American	3 (9.3)
Other	3 (9.3)
ASA Class^a^, n (%)	
I	0 (0.0)
II	19 (51.4)
III	14 (37.8)
IV	4 (10.8)
Follow-up (years)^a^, mean ± SD [range]	4.7 ± 3.2 [0.3–9.7]

*SD* standard deviation, *n* number, *%* percentage, *BMI* body-mass index, *ASA* American Society of Anesthesiologists, ^a^, Calculated based on the total number of procedures (*N*=37) rather than unique patients (*N*=32)

## Results

### Perioperative characteristics

**Fig. 1:**
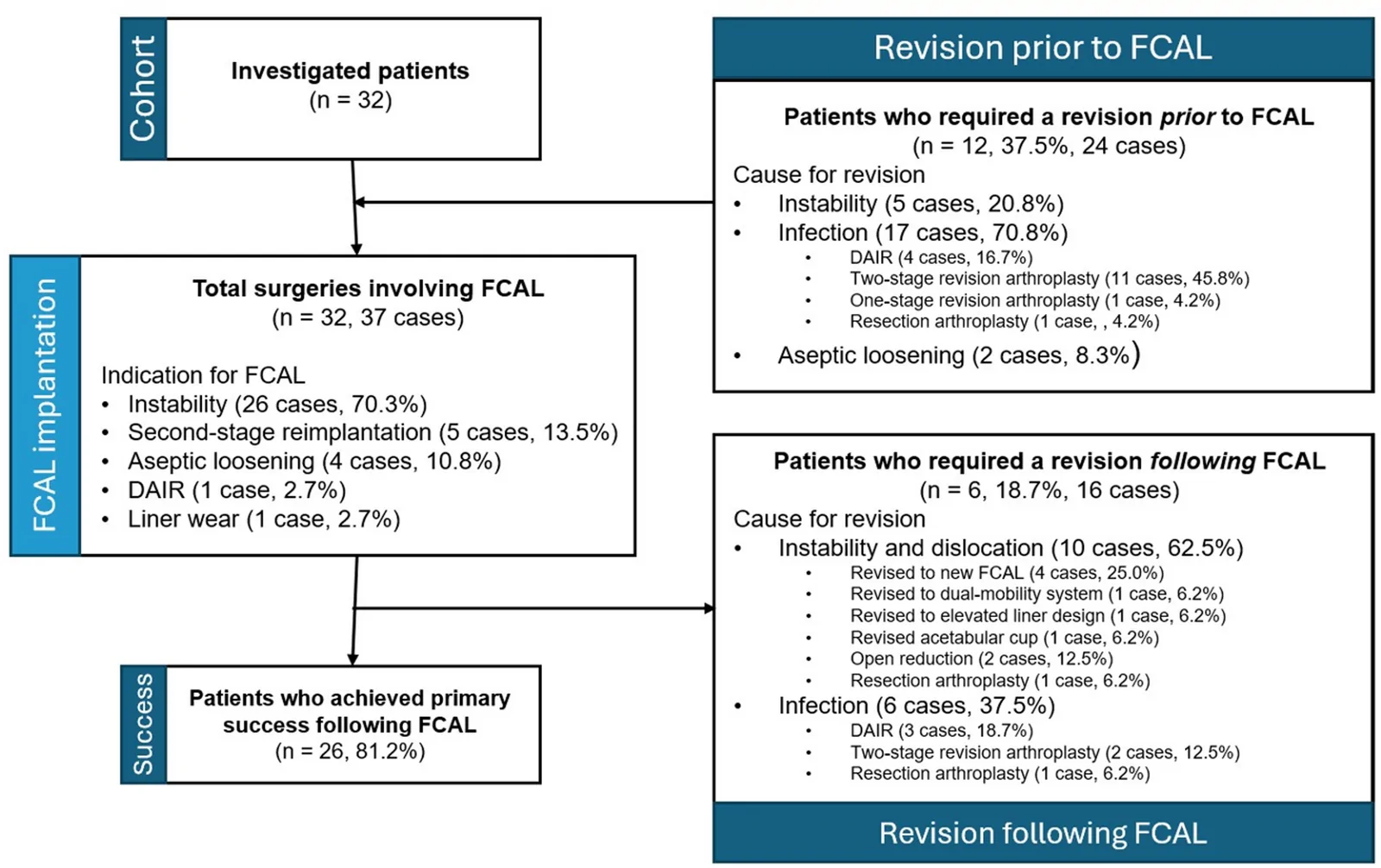
Flow chart demonstrating perioperative characteristics and further reoperation after constrained acetabular liner implantation. *FCAL *Freedom Constrained Acetabular Liner

Of the 37 rTHA procedures utilizing a CAL, the majority had aseptic indications (*n* = 31, 83.8%). Most aseptic cases were performed for instability (*n* = 26, 70.3%), followed by aseptic loosening (*n* = 4, 10.8%) and polyethylene wear (*n* = 1, 2.7%). Six procedures (16.2%) had septic indications: four were second-stage procedures in a planned two-stage exchange and two involved debridement, antibiotics, and implant retention (DAIR) (Fig. 1).

Prior to rTHA with CAL implantation, fourteen procedures involved an existing standard fixed-bearing construct (37.8%), twelve a dual-mobility construct (32.4%), five an existing next-generation CAL (13.5%), four a CAL from a different manufacturer (10.8%), one a static spacer (2.7%), and one a hemiarthroplasty (2.7%).

Across the cohort, 12 patients (37.5%) underwent a total of 24 prior revisions before CAL implantation. Seventeen of these revisions were for infection, five for instability, and two for aseptic loosening. Among the infection-related revisions, eleven were two-stage procedures, four were DAIRs, one was a single-stage revision, and one was a resection arthroplasty (Fig. 1).

In the rTHA procedures analyzed, a CAL was implanted into a pre-existing acetabular cup in most cases (*n* = 26, 70.3%), whereas eleven procedures involved both cup and liner revision (29.7%). Among the 26 cases retaining the cup, the CAL was locked into a compatible modular acetabular component in 24, and cemented into the existing cup in 2.

### Radiographic outcomes

Radiographic evaluation was feasible for 35 of the 37 procedures (94.6%). Preoperative bone defects were most commonly classified as Paprosky Type 1 (77.1%), followed by Type 2 A (14.3%). Postoperative component orientation demonstrated a mean acetabular inclination of 44.3° ± 7.8° and a mean anteversion of 15.9° ± 10.0°. Biomechanical restoration achieved a mean horizontal center of rotation of 36.2 ± 12.5 mm, vertical center of rotation of 21.4 ± 8.5 mm, and femoral offset of 58.5 ± 17.7 mm. For procedures involving full acetabular cup revision, a median of 3 screws (range, 1–5) were utilized for fixation. At latest radiologic follow-up, radiolucent lines were observed in only 1 case (2.9%), with no instances of implant migration or eccentric seating (Table 2).

**Table 2:** Radiographic characteristics and latest follow-up

Parameter	*N* = 35
Preoperative bone defect (Paprosky), n (%)	
1	27 (77.1)
2 A	5 (14.3)
3 A	1 (2.9)
3B	2 (5.7)
Postoperative component orientation, (°), mean ± SD	
Acetabular inclination	44.3 ± 7.8
Acetabular anteversion	15.9 ± 10.0
Biomechanical restoration, (mm) mean ± SD	
Horizontal COR	36.2 ± 12.5
Vertical COR	21.4 ± 8.5
Femoral offset	58.5 ± 17.7
Acetabular fixation (revised cups only, *n* = 11), median (range)	
Number of screws	3 (1–5)
Latest radiologic follow-up, n (%)	
Radiolucent lines	1 (2.9)
Implant migration	0 (0.0)
Eccentric seating	0 (0.0)

*N* number, *%* percentage, *SD* standard deviation, *COR* center of rotation

### Postoperative complications and survivorship

Following CAL implantation, no cases of implant wear, breakage, acetabular cup aseptic loosening, or periprosthetic fracture were observed. However, recurrent dislocation represents a failure of the implant construct. Of the 32 patients in our cohort, as outlined in the Fig. 1 flow diagram, 6 (18.8%) underwent a total of 16 subsequent reoperations – 10 for instability and 6 for infection. Among the 10 instability-related reoperations, 4 involved revision to a new CAL, 2 were open reductions, 1 was revised to a dual-mobility construct, 1 to an elevated liner, 1 to a dual-mobility articulation in a revised cup, and 1 was a resection arthroplasty performed for severe soft-tissue deficiency. Of the 6 reoperations protocols for infection, 3 were DAIR procedures, 2 underwent two-stage exchanges, and 1 was a resection arthroplasty (Fig. 1).

**Fig. 2:**
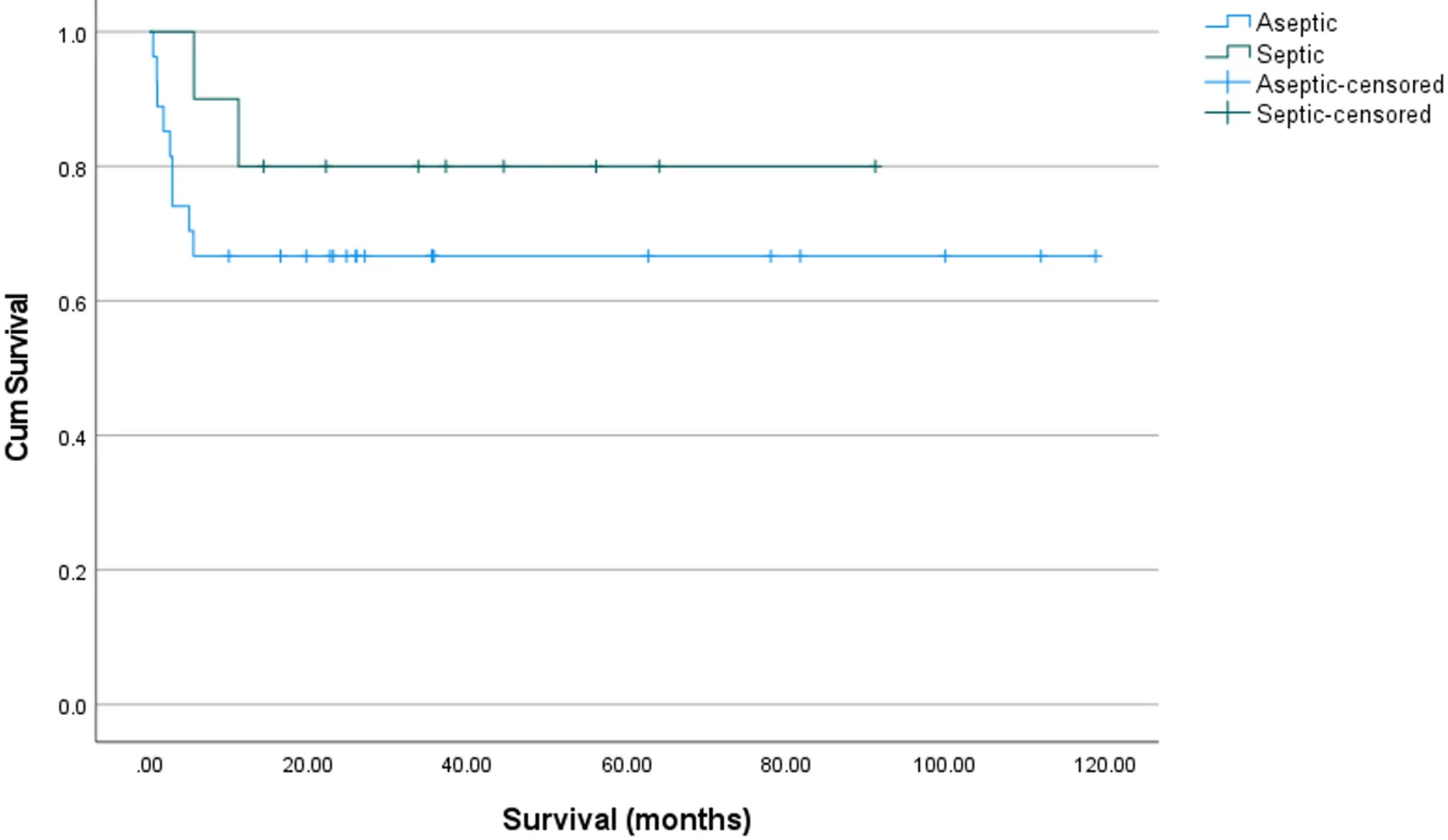
Kaplan-Meier survivorship free from all-cause reoperation after constrained liner reimplantation, stratified by surgical indication. n, number of patients; FCAL, Freedom Constrained Acetabular Liner

Survivorship free from all-cause reoperation was 83.8% at one year and 72.6% at latest follow-up. There was no statistically significant difference in reoperation-free survivorship observed between the aseptic and septic indication groups (*p* = 0.342) (Fig. 2).

## Discussion

Our data demonstrate favorable mid-term survivorship of this CAL in a complex revision THA cohort. Constrained liners have long been used as a salvage option for patients with recurrent instability or abductor mechanism deficiencies [12–15]. However, the shift toward precision arthroplasty demands that these devices do more than universally restrict motion; they must address the specific anatomical and biomechanical deficits of the individual patient. The performance of this next-generation CAL, with an 83.8% reoperation-free survivorship at one year, is comparable to that of other contemporary constrained liners reported in literature. In a recent systematic review, Mancino et al. reported an overall reoperation-free survivorship of 79.9% at latest follow-up in patients treated with constrained liners [12]. Our study therefore expands on existing data and supports the use of this next-generation CAL in complex revision THA as a viable, patient-specific intervention. Despite being outside the scope of this paper, it is worth noting that modern dual-mobility (DM) constructs are a common alternative in high-risk instability cases. However, DM is prone to different rates of complications like intraprosthetic dissociation and liner malseating [24, 25], highlighting the need for CALs. Furthermore, while the 72.6% survivorship is lower than standard revision benchmarks, it must be contextualized within this highly complex revision THA cohort.

Despite generally good outcomes, the use of CALs requires balancing restricted range of motion with the risk of impingement on a patient-by-patient basis. These liners function as mechanical substitutes for compromised soft-tissue support and, as a result, are subjected to mechanical overload and increased stresses at the bone-implant interface. In this context, constrained liners may fail due to aseptic loosening, biomaterial wear, and periprosthetic fracture rather than recurrent instability [14–16, 23]. This risk is exacerbated by repetitive impingement within a reduced arc of motion, which can generate polyethylene fatigue fractures and locking-mechanism failure [15]. Prior studies have reported aseptic loosening incidences between 2 and 7%, with implant wear, breakage, and periprosthetic fracture cumulatively occurring in an additional 2–3% of patients [12, 18, 22]. A previous study specifically evaluating this CAL reported a 1.9% incidence of aseptic loosening, with no cases of liner wear, breakage, or periprosthetic fracture [18].

In our cohort, we observed no cases of implant wear, breakage, acetabular cup aseptic loosening, or periprosthetic fracture. Furthermore, our radiographic analysis of 35 procedures demonstrated excellent preservation of component orientation over time, with only one case of non-progressive radiolucent lines and no cases of implant migration or eccentric seating, supporting this low clinical loosening rate. Notably, this next-generation CAL incorporates liner cut-outs with focal areas of constraint designed to preserve a broad range of motion, allowing surgeons to individually tailor the safe zone to match the patient’s specific spinopelvic kinematics. This personalized modularity potentially reduces stress at the bone-implant interface and contributes to the low incidence of postoperative aseptic loosening, biomaterial wear, and locking-mechanism failure. Larger clinical series with this next-generation CAL, combined with dedicated biomechanical studies focusing on patient-specific modeling, are needed to confirm these findings.

### Limitations

This study is not without limitations. The sample size is inherently limited by the selective use of CALs in only the most complex THAs, limiting the generalizability of our findings. Furthermore, as a tertiary academic referral center, revision procedures account for approximately 2% of our total joint arthroplasty caseload. Even within this high revision burden, this cohort of 32 patients represents a highly select subset of our most complex cases. Because the cohort includes only 6 septic cases, the comparative survivorship analysis between septic and aseptic groups is underpowered and should be interpreted cautiously. Additionally, as a retrospective study conducted at a single high-volume academic center, the study is subject to selection bias, compounded by the exclusion of 16 patients without follow-up, and the results may not be representative of broader patient populations or other practice settings. Although all procedures were performed at one institution by fellowship-trained, high-volume surgeons, variations in surgical technique and postoperative management likely existed and may have influenced outcomes. Furthermore, retrospective data collection limits our ability to fully capture granular, patient-specific variables such as baseline spinopelvic mobility. Additionally, strict radiographic measurements could only be performed on 35 of the 37 procedures due to inadequate imaging, though this represents 94.6% of the cohort. Finally, while patients with 3–4 months of follow-up do not contribute to long-term durability data, their inclusion captures early catastrophic failures. Unlike some prior studies, we did not include patient-reported outcome measures in our analysis [12]. Because recurrent instability severely impacts function and quality of life, the absence of clinical scores is a major limitation. Given the highly individualized nature of these complex cases, our primary objective was to describe implant survivorship and reoperation patterns in a high-risk revision population. Future prospective studies with larger cohorts, standardized patient-reported outcome measures, and longer follow-up are needed to more fully characterize functional outcomes and long-term implant performance.

## Conclusions

This study demonstrates favorable mid-term clinical outcomes and reoperation-free survivorship following complex revision THA using a next-generation constrained acetabular liner system. By offering focal constraint that adapts to individualized soft-tissue and bony deficiencies, this device represents a significant advancement in tailored arthroplasty. Ongoing follow-up and larger multicenter studies will be necessary to determine whether these encouraging mid-term results translate into long-term durability and sustained patient-specific success.

## Data Availability

No datasets were generated or analysed during the current study.
